# Total Arch Replacement with a New Hybrid Device to Manage the Left Subclavian Artery in the Frozen Elephant Trunk Technique

**DOI:** 10.1093/icvts/ivag078

**Published:** 2026-03-10

**Authors:** Leonard Pitts, Markus Kofler, Matteo Montagner, Jörg Kempfert

**Affiliations:** Department of Cardiothoracic and Vascular Surgery, Deutsches Herzzentrum der Charité (DHZC), Berlin 13353, Germany; Charité—Universitätsmedizin Berlin, Freie Universität Berlin and Humboldt-Universität zu Berlin, 10117, Berlin, Germany; DZHK (German Centre for Cardiovascular Research), Berlin, Germany; Department of Cardiothoracic and Vascular Surgery, Deutsches Herzzentrum der Charité (DHZC), Berlin 13353, Germany; Charité—Universitätsmedizin Berlin, Freie Universität Berlin and Humboldt-Universität zu Berlin, 10117, Berlin, Germany; DZHK (German Centre for Cardiovascular Research), Berlin, Germany; Department of Cardiothoracic and Vascular Surgery, Deutsches Herzzentrum der Charité (DHZC), Berlin 13353, Germany; Charité—Universitätsmedizin Berlin, Freie Universität Berlin and Humboldt-Universität zu Berlin, 10117, Berlin, Germany; Department of Cardiothoracic and Vascular Surgery, Deutsches Herzzentrum der Charité (DHZC), Berlin 13353, Germany; Charité—Universitätsmedizin Berlin, Freie Universität Berlin and Humboldt-Universität zu Berlin, 10117, Berlin, Germany; DZHK (German Centre for Cardiovascular Research), Berlin, Germany

## Abstract

**Objectives:** Total arch replacement using the frozen elephant trunk technique remains the gold standard for a definite aortic arch repair. To facilitate surgical management of the left subclavian artery (LSA), a new hybrid frozen elephant trunk device was recently developed.

**Methods:** A 62-year-old female patient presented with acute type A aortic dissection and underwent emergent aortic root and total arch replacement using the novel custom-made Evita Neo EDE hybrid arch device for frozen elephant trunk implantation. To facilitate the management of the LSA, a covered stent connected to the device was inserted into the LSA via guidewire prior to performing the distal anastomosis in zone two.

**Results:** Postoperative computed tomographic angiography demonstrated excellent outcome and technical success with no signs of endoleak or device-related complications. The patient was discharged home 11 days after surgery in a stable clinical condition.

**Conclusions:** The new Neo EDE hybrid arch device for frozen elephant trunk implantation is easy to use and simplifies surgical management of the LSA without adding technical complexity.

## INTRODUCTION

The frozen elephant trunk (FET) technique remains the gold standard for total arch replacement in the case of complex aortic arch pathologies.^1^ Reimplantation of the left subclavian artery (LSA) may represent a critical step of the procedure due to its anatomic localization, especially in the presence of a deep chest or distal origin. A new hybrid device called Evita Neo EDE (Artivion), consisting of a regular FET prosthesis combined with an additional branch for open stenting of the LSA, has been developed recently.

### Device configuration

**Figure 1.**
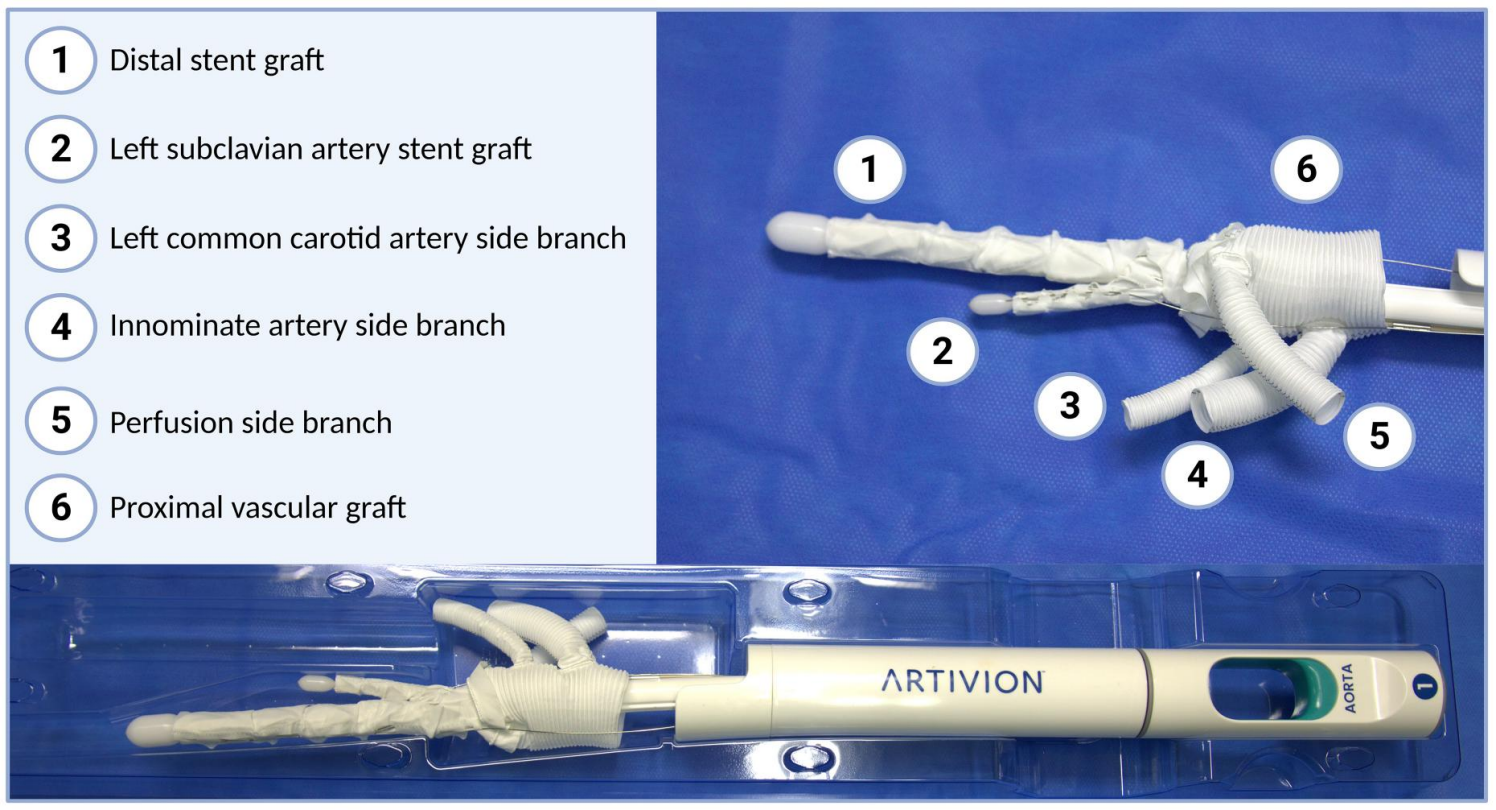
Device Configuration and Overview

The Evita Neo EDE is a custom-made FET device and can be designed in a straight, branched, trifurcated or bifurcated fashion, while a branched prosthesis was used in this case (Figure 1). The FET stent sizing is performed in standard fashion, while the LSA stent graft part is 40 mm long and available with 11 or 15 mm diameter, which will cover artery sizes of 8.5-14 mm.

## CASE PRESENTATION

### Preoperative clinical status and diagnostics

**Figure 2.**
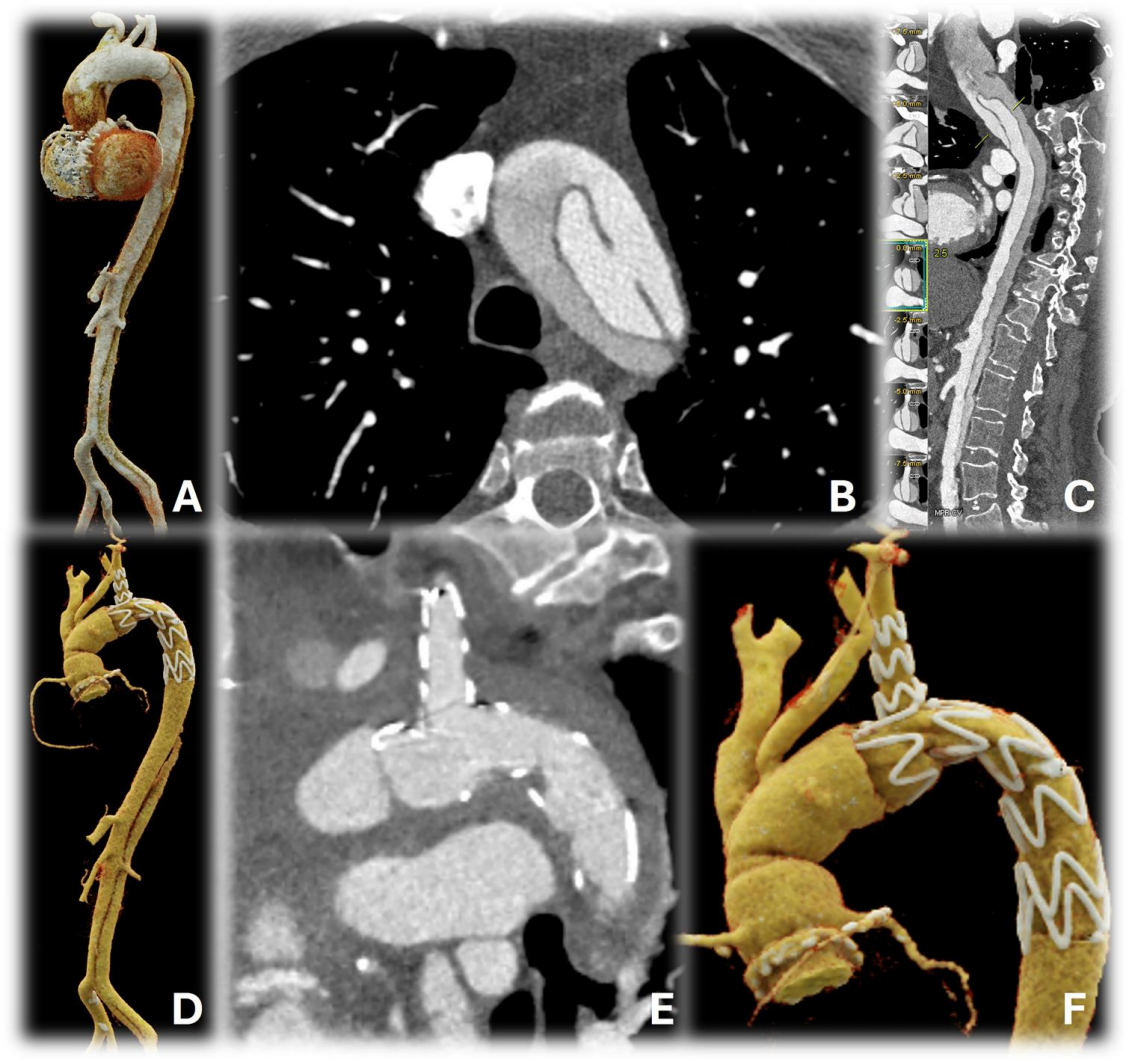
Pre- and Postoperative Computed Tomography Angiography. Preoperative 3D reconstruction of the aorta. Primary entry tear in the distal aortic arch. Centerline reconstruction. Postoperative 3D reconstruction of the aorta. (B) Complete stent unfolding. (C) Satisfactory remodeling and no signs of endoleak

A 62-year-old woman experienced sudden thoracic and back pain while cycling during the morning hours. Immediate computed tomography angiography (CTA) of the aorta revealed an acute type A aortic dissection with a primary tear in the distal aortic arch. The dissection extended from the aortic root to the left common iliac artery (Figure 2A-C). The patient was immediately transferred to our hospital. At admission, the patient was breathing spontaneously and showed stable haemodynamics. There were no signs of clinical or radiological malperfusion. The patient was prepared for emergent aortic root and total arch replacement.

### Surgical procedure and device deployment

A detailed report including all relevant surgical steps is provided in the supplementary material. During distal arrest under moderate hypothermia (28 °C) and bilateral antegrade cerebral perfusion, the ascending aorta and aortic arch were resected until zone 2 before the LSA. A short guidewire (Rosen Wire; Cook Medical) was inserted into the LSA under direct vision in antegrade fashion. Afterwards, the Evita Neo EDE (FET stent 30 mm diameter/130 mm length) was gently pushed into the descending aorta (30 mm diameter) via guidewire, the FET stent was released followed by the side branch stent (11 mm diameter) in the LSA (10.5 mm diameter), which is summarized in Video 1.

## OUTCOMES

The patient was extubated 1 day after surgery and transferred to the intermediate care unit 3 days later (need for non-invasive ventilation) and to the general ward 6 days later. Unfortunately, the patient reported light hypesthesia of the right leg. A cerebral CT followed by cerebral magnetic resonance imaging showed no acute correlations for the reported symptoms, possibly indicating a peripheral nerve affection by femoral cannulation. The patient was discharged home 11 days after surgery after CTA of the aorta (Figure 2D-F), which showed excellent results in terms of technical success and aortic remodelling.

## DISCUSSION

Innovative devices like Rapidlink (Terumo) or FET-FEN (Cook Medical) to simplify management of supra-aortic vessels in terms of FET implantation have just entered the scene of total arch repair and may represent a useful tool to reduce surgical complexity and improve patient outcomes.^2^^,^^3^ The Neo EDE is easy to handle and does not require any specific endovascular skills, while preserving the nature of FET implantation, which is familiar to most aortic surgeons. Compared to classic surgical alternatives like debranching combined with zone 0/1 arch replacement, the advantage lies in non-surgical management of the LSA, which reduces complexity and distal arrest time. Guidewire insertion can also be done in a retrograde manner via the left brachial artery, but it may not be mandatory according to our experience. Folkmann *et al.* reported the first 4 cases with this device, and all patients showed signs of endoleak in the LSA requiring further intervention.^4^ In this case, the LSA diameter was 10.5 mm, matching an 11 mm stent and showed no signs of endoleak. The distance to the left vertebral artery was 4.5 mm, indicating safe use of the device. This should be examined prior surgery, while distances <4 mm, separate origin of the vertebral artery, aneurysm, stenosis and significant kinking of the LSA may be contraindications for this device. However, alternative strategies like in situ anastomosis or extra-anatomic bypass of the LSA may be technically more demanding, time-consuming and carry a higher risk for recurrent laryngeal nerve lesions. Considering the custom-made nature of the device, the chosen prosthesis in this case matched the anatomy of the original patient, who denied surgery. It must be stated at this point that this device should be used with caution in the acute setting as off-label use. However, we believe that frequently used sizes—like in this case—may match a bigger patient population, hopefully leading to an off-the-shelf design in the future.

## CONCLUSIONS

The Evita Neo EDE hybrid arch device provides an intuitive and efficient solution for the FET technique, particularly in managing the LSA, while decreasing operative complexity. More data are needed to confirm the safety and efficacy of this novel device, especially in terms of LSA stent performance.

## Supplementary Material

ivag078_Supplementary_Data

## Data Availability

The data underlying this article are available in the article. Further requests for data can be made to the corresponding author.

## Abbreviations

CTA: Computed tomography angiography
FET: Frozen elephant trunk
LSA: Left subclavian artery
