# Supplementary material for: Total Arch Replacement with a New Hybrid Device to Manage the Left Subclavian Artery in the Frozen Elephant Trunk Technique
Source: Interdiscip Cardiovasc Thorac Surg. 2026 Mar 10;41(3):ivag078. doi: 10.1093/icvts/ivag078 (PMC13098136; doi:10.1093/icvts/ivag078)
Supplement: ivag078_Supplementary_Data [file ivag078_Supplementary_Data.docx]

**Supplemental material**

**Surgical procedure**

Prior to surgery, two invasive arterial lines were placed in the left radial and left femoral artery. Near infrared spectroscopy electrodes were placed bilaterally on the forehead. General anesthesia was continued using propofol and sufentanil. The patient was placed in a supine position prior to standard sterile draping. Simultaneous transesophageal echocardiography showed a preserved biventricular function and moderate aortic valve regurgitation. After application of 2 g cefazoline and team timeout, a skin incision was made in the right infraclavicular fossa. The right axillary artery was exposed while preserving the pectoralis muscle and brachial plexus. After systemic heparinization, arterial cannulation using an 18Fr cannula was performed via seldinger technique. Venous cannulation was performed percutaneously via the right femoral vein under ultrasound guidance with a 25Fr cannula, followed by initiation of cardiopulmonary bypass and careful systemic cooling to approximately 31°C considering the risk of ventricular fibrillation prior placement of a left ventricular vent. An additional guidewire was placed over the left common femoral artery into the true lumen of the descending aorta for later stent deployment. After median sternotomy, the innominate vein as well as the aortic arch including the brachiocephalic trunk and left common carotid artery were carefully exposed. The pericardium was opened, and a left ventricular vent was inserted via the right pulmonary vein and cooling was continued. The ascending aorta was clamped, incised and DelNido cardioplegia was administered directly in both coronary ostia, which was repeated once after 60 minutes. Both the ascending aorta and the aortic root (no root entry, 34mm diameter but deep dissection reaching the annular plane) were resected, the coronary ostia isolated as buttons and after sizing, a 23mm “Konnect” biological valved conduit (Edwards Lifesciences, Irvine, CA, USA) was selected for implantation. Reaching 28°C core temperature (bladder + nasopharyngeal), the flow was reduced to 10ml/kgKG/min for selective antegrade cerebral perfusion. The innominate and left common carotid artery were clamped, the aortic cross clamp removed, and the aortic arch was resected until zone two prior the LSA. An additional perfusion tip catheter was inserted into the left common carotid artery for bilateral antegrade cerebral perfusion. A short guidewire (Rosen Wire; Cook, Bloomington, IN, USA) was inserted into the LSA under direct vision in antegrade fashion. Then, the Evita Neo EDE (FET stent 30mm diameter / 130mm length) was gently pushed into the descending aorta (30mm diameter) via guidewire, the FET stent was released followed by the side branch stent (11mm diameter) in the LSA (10.5mm diameter), which is summarized in Video 1. After performing the distal anastomosis between the aorta and the FET collar, a cannula was placed in the perfusion branch of the prosthesis. Systemic perfusion and rewarming were initiated (distal arrest time 26 minutes) and the left common carotid artery was anastomosed to the corresponding side branch. Consequently, the chosen conduit was implanted using pledget-reinforced mattress sutures in everting technique according to Bentall-deBono followed by the proximal anastomosis. After careful de-airing, systemic perfusion was regained after 115 minutes of cross-clamp time. Finally, the innominate artery was anastomosed to the last corresponding side branch. Reperfusion, de-cannulation, meticulous hemostasis as well as thorax and wound closure were uneventful (procedural time 269 minutes). The patient was transferred to the intensive care unit under stable hemodynamics.
